# Mechanical and Microstructural Performance of UHPC with Recycled Aggregates Modified by Basalt Fiber and Nanoalumina at High Temperatures

**DOI:** 10.3390/ma18051072

**Published:** 2025-02-27

**Authors:** Hong Jiang, Liang Luo, Yuan Hou, Yifei Yang

**Affiliations:** 1 Anhui Water Conservancy Technical College, Hefei 231603, China; ahsdxy_jianghong@163.com; 2 School of Transportation and Science Engineering, Harbin Institute of Technology, Harbin 150090, China; 3 School of Civil Engineering, Yancheng Institute of Technology, Yancheng 224051, China; houy487786@163.com (Y.H.); 17839613163@163.com (Y.Y.)

**Keywords:** high-temperature performance, crack resistance, basalt fiber, nanoalumina, recycled aggregates, digital image correlation, mechanical properties, microstructure

## Abstract

This study investigates the mechanical properties and microstructure of basalt fiber (BF) and nanoalumina (NA)-modified ultra-high-performance concrete with recycled aggregates (UHPC-RA) under high-temperature conditions. The effects of different replacement rates of recycled aggregates (RAs), BF content, and NA content on the compressive strength, splitting tensile strength, and elastic modulus were evaluated at ambient temperatures and after exposure to 200 °C, 400 °C, 600 °C, and 800 °C. The results show that mechanical properties decrease with temperature rise, but specimens containing BF exhibited improved crack resistance and better high-temperature integrity. The incorporation of NA enhanced the thermal stability and heat resistance of the concrete. Digital image correlation (DIC) was used to monitor real-time surface deformation, and scanning electron microscopy (SEM) analysis revealed improved microstructure with reduced porosity and cracks. This study demonstrates that the combination of BF and NA significantly enhances the high-temperature performance of UHPC-RA, which holds promising potential for applications in environments subjected to elevated temperatures.

## 1. Introduction

With the continuous development of the global construction industry, the performance requirements of building materials are constantly increasing. Traditional concrete materials have exposed some significant problems in actual use, such as poor durability, low strength and ability to be easily affected by the environment [1,2]. In order to overcome these problems, the development of building materials with higher performance, environmental friendliness and economy has become the main direction of modern building material research [3]. Ultra-high-performance concrete (UHPC) has been widely used in the construction field because of its excellent mechanical properties, durability and high temperature resistance [4]. However, the high production cost of UHPC has limited its popularity in large-scale projects. In order to reduce costs and further improve its overall performance, researchers are increasingly turning to new materials such as recycled aggregate (RA) [5], basalt fiber (BF) [6] and nanomaterials (NAs) [7] to improve the mechanical and durability of UHPC.

In the context of promoting sustainable development, the use of RA to replace natural aggregates has become an effective way to reduce the environmental burden of concrete production [8]. RA is usually derived from construction waste, and its physical and chemical properties are different from natural aggregates, so the use of RA may reduce the mechanical properties of concrete. However, recent studies have shown that the mechanical properties and durability of recycled aggregate concrete (RAC) can be significantly improved through reasonable selection of RA, optimization of the concrete mix ratio and application of modification technology [9,10]. For example, surface treatment, mineral admixtures and composite materials have made significant progress in improving the performance of RA concrete [4].

In many construction projects, concrete is often exposed to high-temperature environments, and its fire resistance becomes particularly crucial in the event of a fire. High temperatures lead to moisture evaporation and an increase in porosity, which adversely affects the mechanical properties, toughness, and durability of concrete [11]. Therefore, understanding the effects of high temperatures on the performance of UHPC is essential. Existing studies [12] indicate that under high-temperature conditions, the mechanical properties of UHPC, such as compressive strength and flexural strength, significantly decrease. However, optimizing its composition or incorporating modified materials can effectively improve its high-temperature resistance. For example, the addition of mineral admixtures, polymers, and fiber materials can enhance the crack resistance, thermal shock resistance, and fire resistance of UHPC. The application of RA in UHPC, especially in high-temperature environments, has also gained attention and demonstrated significant potential in improving the durability and mechanical properties of concrete [13,14]. Moreover, in recent research on fiber-reinforced concrete (FRC), non-destructive evaluation (NDE) techniques, particularly acoustic emission (AE) and ultrasonic pulse velocity (UPV), have been widely used in assessing fire-induced concrete damage. These techniques can monitor crack propagation and damage evolution in real-time without causing additional harm to the structure, making them highly advantageous for evaluating high-temperature damage. Specifically, AE technology plays a critical role in studying crack propagation and bridging effects in fiber-reinforced concrete under high-temperature conditions. For example, ref. [15] explored the thermal damage behavior of self-compacting high-strength fiber-reinforced concrete (SCHSFRC) and self-compacting high-strength concrete (SCHSC), showing that fibers effectively bridge heat-induced cracks and improve the high-temperature performance of the concrete. Additionally, ref. [16] investigated the application of AE in assessing fire damage in steel fiber-reinforced concrete (SFRC), emphasizing the correlation between AE parameters and mechanical properties, thereby confirming the significant potential of AE for assessing post-fire structural integrity. Recent studies indicate that combining AE and UPV NDE techniques can not only accurately monitor crack propagation but also effectively identify fire-induced damage in concrete, further revealing the damage mechanisms and recovery capabilities of fiber-reinforced concrete under high temperatures. Therefore, the application of NDE techniques in fire damage assessment provides strong support for practical engineering, offering real-time and precise damage monitoring and evaluation.

Over the past decade, much research has focused on improving the high-temperature performance of UHPC. These studies have investigated the effects of various materials (including traditional fibers and nanomaterials) on the mechanical properties of UHPC at high temperatures [17,18]. However, most of these studies have focused on a single type of fiber or nanomaterial, or have considered the effects of regenerated aggregates (RAs) alone, without addressing their combined effects at high temperature exposure [19]. The addition of BF has been shown to improve crack resistance and mechanical strength at high temperatures due to its high thermal stability and good bonding with the cement matrix [20]. On the other hand, NA contributes to the densification of the cement matrix and enhances the thermal stability of the material by reducing the formation of micro-cracks [21]. However, little attention has been paid to the synergistic effects of BF and NA binding, especially in UHPC systems containing RA, which are known to have poor thermal stability due to degradation of the interfacial transition zone (ITZ) at high temperatures [22].

As a new type of natural fiber material, BF has been widely used in concrete due to its excellent mechanical properties, high-temperature resistance, and corrosion resistance [6]. Studies show that BF enhances the tensile strength, crack resistance, and toughness of concrete, as well as its high-temperature performance. BF improves the microstructure of concrete by reducing crack formation, thereby enhancing its durability [23]. Additionally, BF has a positive impact on the properties of RAC, strengthening its fire resistance and mechanical characteristics. As such, the incorporation of BF is considered an effective method to improve the overall performance of UHPC. With advances in nanotechnology, the application of nanomaterials in concrete has become increasingly widespread [24]. Nanoalumina (NA), a high-performance nanomaterial, has significant potential to enhance the strength, durability, and high-temperature performance of concrete. Research [25] indicates that NA refines the hydration products of concrete, improving its microstructure, density, and impermeability. Furthermore, NA enhances the thermal stability of concrete, improving its performance in high-temperature environments. Therefore, the application of NA in UHPC, particularly under high-temperature conditions, demonstrates considerable potential.

The incorporation of RA into concrete under high-temperature conditions has gained increasing attention. Existing studies [26,27,28] indicate that RAC exhibits good mechanical properties, seismic resistance, and fire resistance, and has been successfully applied in practical engineering projects [29,30]. Khan et al. [31] investigated the performance of RAC with different RA replacement rates (0%, 30%, 50%, 70%) under high temperatures ranging from 250 °C to 500 °C, finding that the mechanical properties remained stable and were not significantly affected. However, when the temperature exceeded 500 °C and the RA replacement rate exceeded 30%, both the elastic modulus and Poisson’s ratio were significantly impacted. Additionally, the study proposed a residual strength factor to predict the residual strength of RAC after exposure to high temperatures. Chen et al. [32] demonstrated experimentally that as temperature increased, the appearance, mass loss, and mechanical properties of recycled concrete changed. Strength showed a negative correlation with temperature, while elastic modulus and peak stress gradually decreased with increasing temperature, with more pronounced degradation. Algourdin et al. [33] indicated that high temperatures induce decomposition of cement hydration products and mismatches in thermal expansion between the aggregates and the hardened paste, leading to paste delamination and subsequently affecting concrete performance. Although these studies provide important data on the performance of RAC under high temperatures, there is still a lack of comprehensive research on the high-temperature performance of UHPC with the addition of RA, particularly regarding the mechanical properties and behavior under temperature effects after incorporating modified materials (fibers and nanomaterials) in fire conditions. Specifically, studies on the synergistic effects of modified materials, such as BF and NA, under high-temperature conditions are still limited. Existing research mainly focuses on the impact of individual modified materials on high-temperature performance, with few studies considering the combined effect of BF and NA on the high-temperature performance of UHPC-RA. Moreover, the current literature has not sufficiently explored the comprehensive effects of different RA replacement rates, BF content, and NA content on the mechanical properties and microstructure of UHPC-RA under high-temperature conditions. Additionally, how to reveal the behavioral mechanisms of these materials under high-temperature conditions through microstructural analysis remains an unresolved issue. Therefore, the research question of this study is as follows: How can the composite modification of UHPC-RA using BF and NA improve its mechanical properties under high-temperature conditions, and how can the changes in microstructure further explain this mechanism?

This study aims to investigate the effects of BF and NA on the mechanical properties of recycled aggregate ultra-high-performance concrete (UHPC-RA) under high-temperature conditions. Specifically, Section 2 will provide detailed information on the experimental design and material preparation, including the concrete mix proportions, experimental conditions, and testing methods. Section 3 will present the experimental results, with a focus on analyzing the effects of different RA replacement rates, BF contents, and NA contents on the mechanical properties of concrete under high-temperature conditions. Additionally, the microstructural changes in the concrete will be analyzed using scanning electron microscopy (SEM) and digital image correlation (DIC) techniques, and the mechanisms affecting high-temperature performance will be discussed. Section 4 will summarize the research findings and discuss their potential applications in practical engineering and future research directions.

## 2. Methodology

### 2.1. Material Selection

This study uses seven primary materials to prepare UHPC-RA, categorized into three groups: powder binders, aggregate fillers, and fiber reinforcement materials.

**Figure 1:**
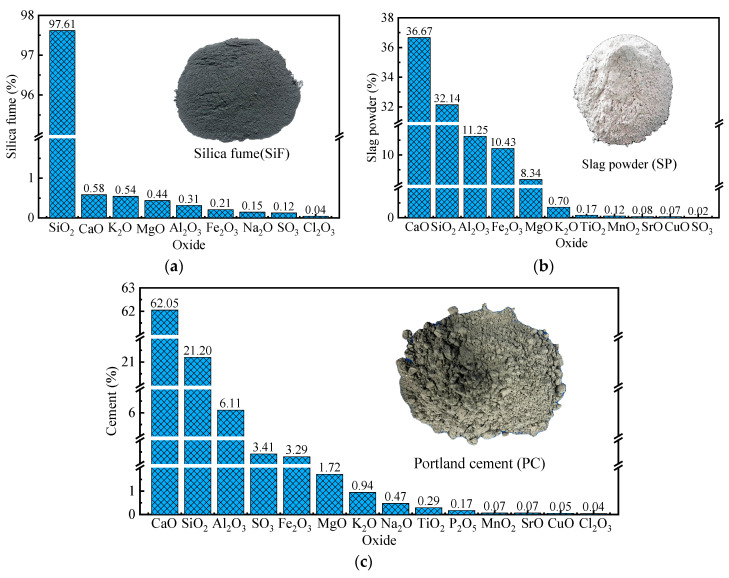
Chemical composition of cementitious materials: (**a**) silica fume; (**b**) slag powder; (**c**) cement.

- Powder binders: These include silica fume (SiF), slag powder (SP), Portland cement (PC), and nanoalumina (NA). PC serves as the primary binder, filling millimeter-scale gaps between aggregates. Micron-scale particles (SiF and SP) fill the gaps between micro-level particles, and nano-scale particles (NA) further enhance the packing density. This combination of materials optimizes the packing density of the concrete mix.
- Aggregate fillers: These consist of fine aggregates (natural river sand), coarse aggregates (natural aggregates, A), and recycled aggregates (RAs).
- Fiber reinforcement: Basalt fiber (BF) is used to enhance the mechanical performance of the concrete.

(1) Cement and Additives

- Silica Fume (SiF): Silica fume is sourced from Changsha, Hunan Province, and serves as a supplementary material for pozzolanic reactions. It fills voids between cement particles, enhancing early strength and durability. The SiO_2_ content is 98% by mass, and the chemical composition is shown in Figure 1a.
- Slag Powder (SP): SP is finely ground blast furnace slag with a specific surface area of 4360 cm^2^/g and a 28-day activity index of 95%. It improves the workability of concrete. Its primary chemical composition is depicted in Figure 1b.
- Cement (PC): The cement used in this study is a Type II 52.5-grade Portland cement with chemical compositions as shown in Figure 1c. It contains 62.05% CaO and 21.2% SiO_2_, with a Blaine specific surface area of 3580 cm^2^/g and an average particle size of 15.3 μm.

Physical properties of these three binder materials are detailed in Table 1, and the particle size distribution of each powder binder is shown in Figure 1a. 

**Figure 2:**
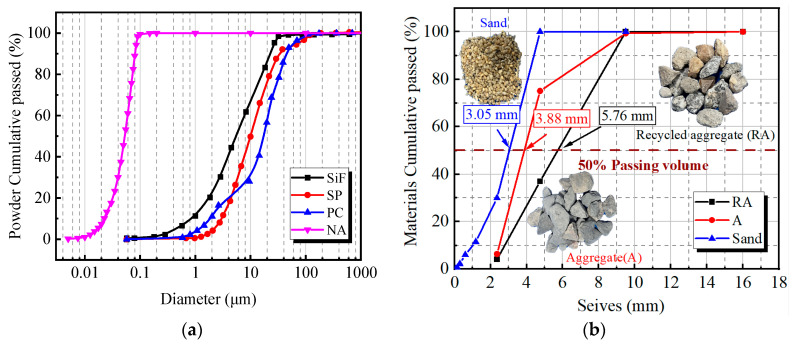
Grading curves: (**a**) powder material grading; (**b**) aggregate grading.

(2) Aggregates

- Fine Aggregate: The fine aggregate used is natural river sand, with a particle size range of 0–3.6 mm.
- Coarse Aggregate: The coarse aggregates consist of natural basalt gravel (A) with a size range of 5–16 mm and recycled aggregate (RA) with the same particle size range, sourced from crushed original C50 concrete specimens. The basic properties of A and RA are shown in Table 2. Figure 2b illustrates the irregular edges, minute pores, and old cement mortar on the RA, indicating inherent damage and defects. The particle size distribution of fine and coarse aggregates is shown in Figure 2b, where the materials’ average particle size is comparable at the 50% passing rate, suggesting that the mixture will achieve dense packing. Before mixing, the RA undergoes a series of pre-treatment measures to ensure it reaches an optimal condition. Firstly, the RA is cleaned using high-pressure water to remove surface-attached cement paste and impurities, followed by mechanical screening to eliminate any oversized or non-compliant particles. Next, the RA is soaked in water for 24 h to reduce its water absorption rate, preventing any negative impact on the water-to-cement ratio of the concrete. Finally, the soaked RA is air-dried to achieve a Saturated-Surface-Dry (SSD) condition, ensuring no excess surface moisture while maintaining internal water saturation. This pre-treatment process effectively reduces water absorption and residual cement paste issues in RA, thereby preventing any adverse effects on the strength and durability of the concrete.

**Table 2:** Properties of various treated and untreated recycled aggregates.

Parameter	Specific Gravity (kg/m^3^)	Elongation Particle (%)	Flatness Particle (%)	Water Absorption (%)	Aggregate Impact Value (AIV)	Abrasion Loss (%) (LA)
Standard methods	BS812, Part 2 [34]	ASTM D4791 [35]	ASTM D4791 [35]	BS812, Part 2 [34]	BS812, Part 112 [36]	ASTM C131 [37]
A	2650	10	25	0.7	12	20
RA	2380	16	17	1.90	20	45

(3) Basalt Fiber

Basalt fiber (BF) used in this study is a 12 mm chopped fiber produced by Changsha Building Materials Co. It has a tensile strength of 1050 MPa, and its key properties are listed in Table 3. BF exhibits excellent high-temperature resistance, ablative resistance, and thermal stability, with an operational temperature range from −269 to 960 °C. At 400 °C, its post-failure strength retains 85% of its original value, and at 600 °C, it retains 80%. The softening point is 960 °C, and the melting point is 1450 °C.

(4) Nanoalumina

**Figure 3:**
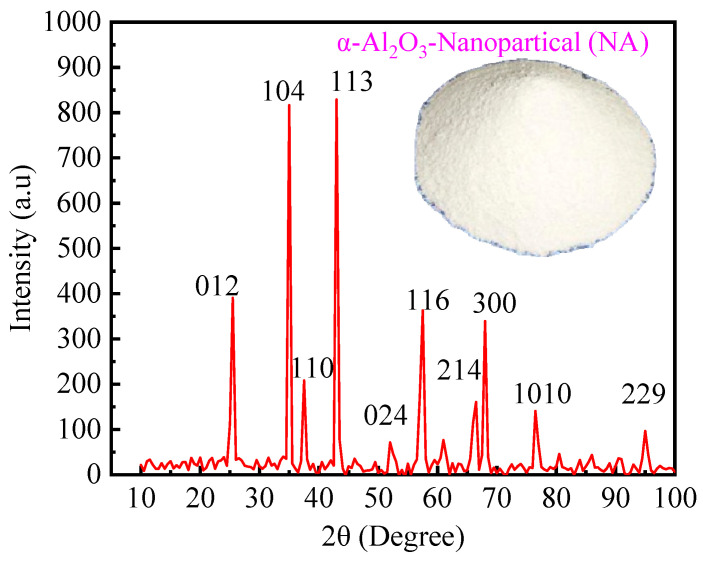
XRD pattern of nanoalumina.

NA particles, as an excellent modifier for concrete, have small particle sizes (typically 30–40 nm) and a large specific surface area (20 m^2^/g). These properties allow NA to fully react with cement particles and water molecules, improving the strength and durability of the concrete. NA also enhances the thermal stability of concrete, making it more heat-resistant in high-temperature environments, especially during fire or extreme conditions. Additionally, NA fills the microvoids in concrete, reducing moisture and gas permeability, significantly improving its impermeability. NA reacts with hydration products in cement, forming more calcium silicate hydrate (C-S-H) to increase the density and strength of the concrete. NA particles (TAP-A21) were supplied by Nanjing Tianxing Materials Co. (Nanjing, China), and their average particle size and physical properties are given in Table 4. The X-ray diffraction (XRD) pattern of α-phase NA is shown in Figure 3, with characteristic peaks at d-values of 0.2085, 0.2551, 0.1601, 0.3480, and 0.2379 nm, and corresponding 2*θ* values of 43.36°, 35.15°, 57.50°, 25.58°, and 37.78°. α-Al_2_O_3_ belongs to the trigonal crystal system and is the most stable phase, with a high melting point and mechanical strength.

### 2.2. Experimental Design and Grouping

To investigate the mechanical properties of UHPC-RA at different temperatures, the experiment focuses on the effects of RA replacement rate, fiber content, and temperature variation on the compressive strength, splitting tensile strength, and elastic modulus of UHPC-RA after high-temperature exposure. RA replacement rates of 0%, 50%, and 100% are used to substitute natural aggregates in the concrete mix. BF content is changed to 0 kg/m^3^, 1 kg/m^3^, and 2 kg/m^3^. NA is added as a substitute for cement, in amounts of 0%, 3%, and 5% by total binder mass. The study includes seven experimental groups, with specific mix proportions as shown in Table 5. The sample labels represent the RA replacement rate and BF content, e.g., R100B2A5 indicates 100% RA replacement, 2 kg/m^3^ BF, and 5% NA. For each mix, 210 cubic specimens (100 mm side length) and 105 prism specimens (100 mm × 100 mm × 300 mm) are prepared. The cubic specimens are used to test the compressive and splitting tensile strengths, while the prism specimens are used to measure the elastic modulus.

Additionally, 1.67% of a high-performance water reducer (HPWR) is added. The HPWR (ZY-S model) supplied by Hunan Zhongyan Building Materials (Hunan, China) is a polycarboxylate-based liquid, light yellow or transparent, with a pH of 6–8, free of chlorides, low in alkali, a solid content of 25%, and a water-reducing rate of 25–40%. Physical properties and its effect on concrete are detailed in Table 6.

### 2.3. Sample Preparation

**Figure 4:**
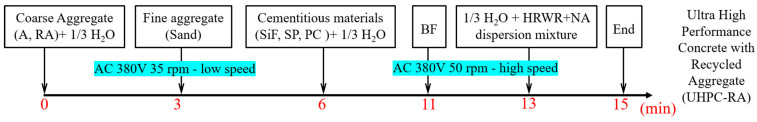
Mixing procedure and mixer operation parameter.

Prior to specimen preparation, workability testing was conducted using the standard testing method for concrete mix performance in accordance with GB/T50080-2016. The slump and slump flow were found to be within the ranges of 154–245 mm and 306–580 mm, respectively. The preparation process follows the steps shown in Figure 4:

1. At low speed, coarse aggregates and one-third of the water are added (0–3 min). Sand is then added, and mixing continues (3–6 min).
2. The remaining powder binders and the other third of the water are added (6–11 min).
3. The mixer is adjusted to high speed (50 rpm), and BF is gradually added (11–13 min), followed by the high-performance water reducer and the last third of the water (13–15 min). The mixing continues until the mixture is homogeneous.
4. The fresh mixture is poured into molds and compacted on a vibration table to eliminate air bubbles. After 24 h, the specimens are demolded and cured in a curing chamber at 20 ± 2 °C and 90–95% relative humidity for 28 days.

### 2.4. High-Temperature Exposure Test

**Figure 5:**
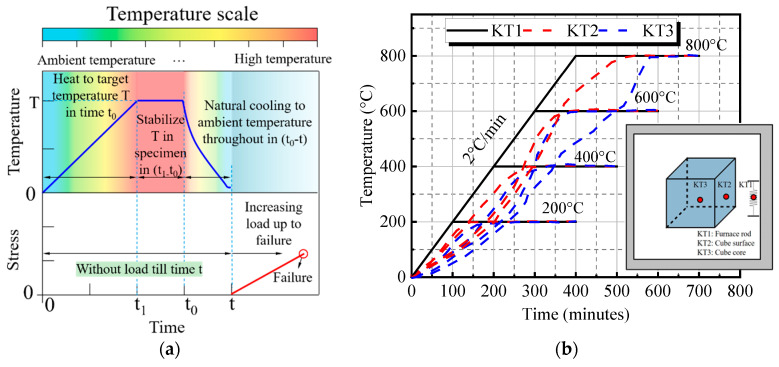
Temperature history of the heated sample: (**a**) residual state heating loading scheme; (**b**) temperature evolution in the furnace and in the different parts (core/surface) of the sample.

To investigate the high-temperature performance of UHPC-RA, specimens are subjected to thermal treatment in a TDL-1400F high-temperature furnace. Based on previous studies [39,40], the exposure temperatures are set at 25 °C (control group), 200 °C, 400 °C, 600 °C, and 800 °C, totaling five temperature levels. As shown in Figure 5a, the specimens are heated at a rate of 2 °C/min to the target temperature, held at a constant temperature for 5 h to ensure uniformity, and then naturally cooled to room temperature before mechanical testing. The 5 h holding time can ensure that the concrete sample is fully affected by heat and can achieve thermal stress balance and thermal stability; this is consistent with existing research work [41,42]. Figure 5b shows the temperature–time curves measured by K-type thermocouples at three key locations: the heating silicon carbide rods (KT1), the specimen surface (KT2), and the specimen center (KT3). The temperature in the center (KT3) takes slightly longer to reach the target compared to the surface (KT2). Due to the difference in heat conduction between the inside and outside of the concrete material and the existence of temperature gradients, the surface of the specimen is more likely to rapidly heat up, while the central part requires a longer time to reach the same temperature. Overall, it still conforms to the heating regime. After high-temperature treatment, the specimens are cooled to room temperature, and the mass loss is measured. The mass loss rate is calculated based on the change in mass before and after high-temperature treatment, with the average value taken for each group of specimens.

### 2.5. Mechanical Property Testing Methods

(1) Compressive Strength and Splitting Tensile Strength

Compressive and splitting tensile strengths are tested according to GB/T 50081-2019 [43], using 100 mm cubic specimens. Loading is applied perpendicular to the casting surface using a WAW-500-G electro-hydraulic servo testing machine. The loading rate for compressive strength and elastic modulus tests is 0.5 MPa/s, while the splitting tensile strength test is conducted at a rate of 0.05 MPa/s.

(2) Elastic Modulus

The elastic modulus is tested according to GB/T 50081-2019 [43] using 28-day cured concrete prism specimens (100 mm × 100 mm × 300 mm). Static testing is performed under compression to determine the elastic modulus.

### 2.6. Microstructure Analysis: Scanning Electron Microscope (SEM)

After mechanical testing, small samples are cut for microstructure analysis. Samples are pretreated by immersion in alcohol to halt hydration, and then placed in a vacuum oven to remove residual alcohol. The treated samples are coated with gold and evacuated to enhance electrical conductivity. Microstructural observation is performed using a JSM-IT500LV scanning electron microscope (SEM) at an accelerating voltage of 20 kV.

### 2.7. Application of DIC Technology

(1) Principle of DIC Technology

Digital image correlation (DIC) technology provides full-field, non-contact measurement of strain and displacement during loading by tracking target points on the specimen’s surface. The principle of DIC is based on monitoring the coordinate changes of target points before and after deformation, allowing for real-time, full-field observation of the specimen’s strain and displacement during the loading process [44]. The calculation of the target point before and after deformation is shown in Equations (1)–(3):

(1) $$C_{f,g}(\bar{P})=\sum_{x=-M}^{M}\sum_{y=-M}^{M}\left[\frac{f(x,y)-f_{m}}{\sqrt{\sum_{x=-M}^{M}\sum_{y=-M^{\prime }}^{M}{\left[f(x,y)-f_{m}\right]}^{2}}}\right]-\left[\frac{g\left(x^{\prime },y^{\prime }\right)-g_{m}}{\sqrt{\sum_{x=y-M^{\prime }}^{M^{\prime }}\sum_{y=-M^{\prime }}{\left[g\left(x^{\prime },y^{\prime }\right)-g_{m}\right]}^{2}}}\right]$$

(2) $$f_{m}=\frac{\sum_{x=-M}^{M}\sum_{y=-M^{\prime }}^{M}{\left[f(x,y)\right]}^{2}}{{\left(2M+1\right)}^{2}}$$

(3) $$g_{m}=\frac{\sum_{x=y-M^{\prime }}^{M^{\prime }}\sum_{y=-M^{\prime }}{\left[g\left(x^{\prime },y^{\prime }\right)-g_{m}\right]}^{2}}{{\left(2M+1\right)}^{2}}$$

where *f*(*x*, *y*) and *g*(*x’*, *y’*) represent the grayscale values of the source and target sub-regions’ coordinates before and after deformation; *f_m_* and *g_m_* are the global average values in the deformed image; *M* is the local coordinate of each data point in the local displacement field; and $C_{f,g}(\bar{P})$ for the local deformation difference is determined by the correlation coefficient of the corresponding regions in the source and target images, quantifying the similarity between the source and target regions, with values ranging from 0 to 1.

(2) Application to Concrete Mechanical Performance Testing

**Figure 6:**
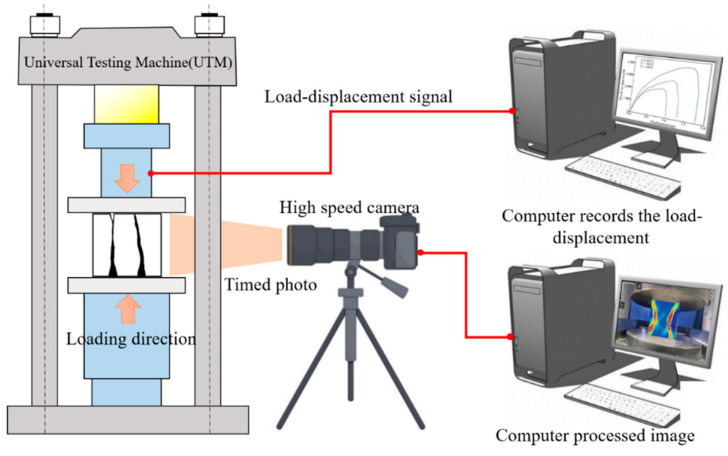
Schematic diagram of DIC monitoring system.

DIC is an optical method for measuring surface deformation. This technology tracks the displacement vectors of surface feature points during deformation, thereby computing the full-field displacement and corresponding strain of the specimen. This allows for the observation of crack formation and propagation. To ensure clear images and accurate analysis, precise experimental preparation is required. As shown in Figure 6, the loading side of the specimen is first treated with white paint, and small black dots are randomly arranged in the region of interest. The data acquisition process is carried out by a system composed of a high-speed camera, a universal testing machine (UTM), an image acquisition card, and a computer. The system captures images at regular intervals and performs subsequent calculations.

## 3. Results and Discussion

### 3.1. Visual Inspection and Mass Loss

**Figure 7:**
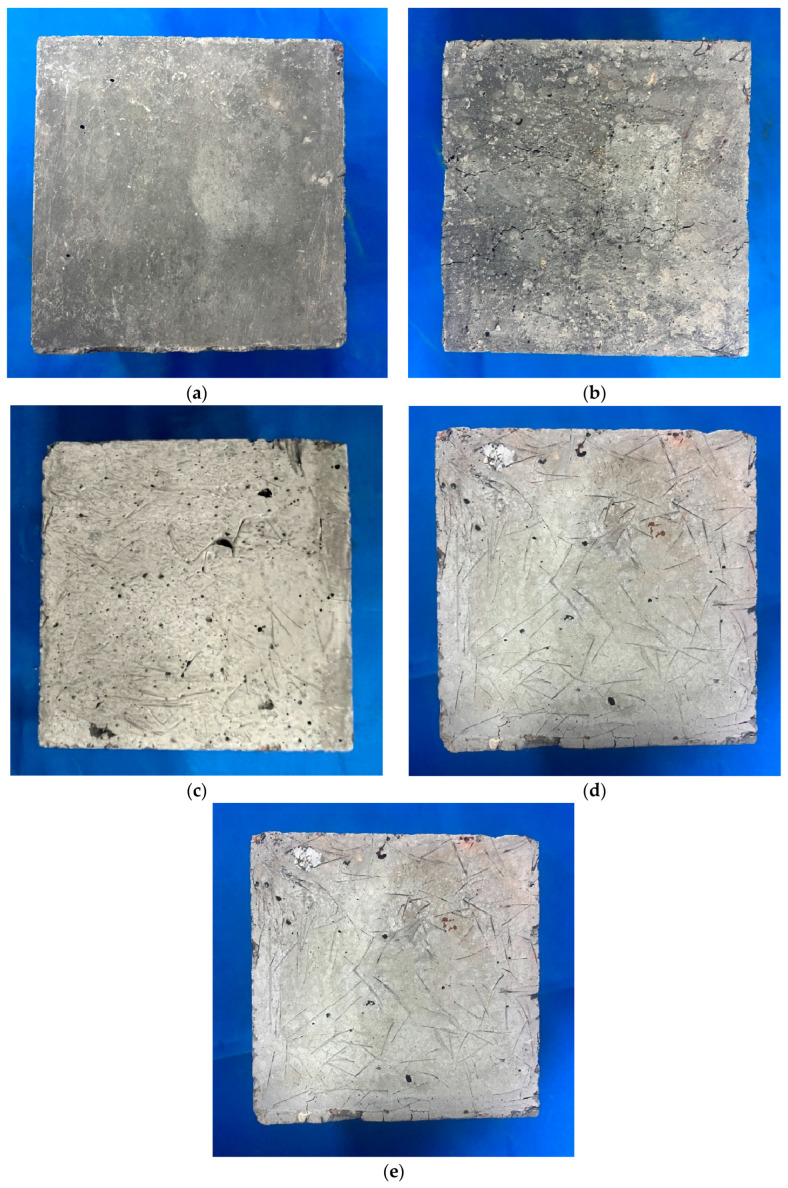
The appearance and color changes of BF-reinforced UHPC-RA at the following temperatures: (**a**) 25 °C; (**b**) 200 °C; (**c**) 400 °C; (**d**) 600 °C; (**e**) 800 °C.

Figure 7 shows the significant visual changes in BF-modified UHPC-RA concrete after exposure to various temperatures. At ambient temperature, the concrete has a bluish-gray color, and the specimen is intact with no cracks. As the temperature increases, the color of the concrete gradually changes: at 200 °C, the surface turns slightly yellow, but no significant cracks appear; at 400 °C, the color darkens, and fine cracks begin to appear; at 600 °C, the color lightens further, and cracks increase; at 800 °C, the surface turns gray-white, with a noticeable increase in cracks and surface peeling. At different temperatures, the specimen’s color changes from blue-gray to gray-white, accompanied by a significant reduction in crack resistance. At 400 °C, microcracks appear, and at 800 °C, the cracks increase, forming a zigzag pattern, with some holes also observed. However, with increasing BF content, crack resistance improves, and the initiation temperature for cracks decreases. Specimens with high BF content (e.g., R0B2A0, R100B2A0) showed no significant cracking at high temperatures, while specimens with low BF content (e.g., R0B0A0) began to crack at 800 °C, with a larger number of jagged cracks. This indicates that the introduction of BF effectively suppressed crack propagation, enhancing the high-temperature integrity of the concrete. Overall, BF incorporation improves the crack resistance of concrete, particularly in high-temperature environments, helping to maintain structural stability.

As the temperature increases, internal moisture evaporation and high-temperature decomposition of hydration products occur within the specimen [45]. UHPC-RA, with its inclusion of RA, exhibits characteristics such as porosity and a higher water-to-cement ratio, which leads to a significantly different mass loss rate compared to conventional concrete at high temperatures. Therefore, it is essential to study the mass loss rate of UHPC-RA after exposure to high temperatures. The mass loss rate is calculated using Equation (4):

(4) $$\Delta W=\frac{m_{0}-m_{1}}{m_{0}}\times 100\%$$

where *m*_0_ is the original mass of the specimen before high-temperature exposure, and *m*_1_ is the residual mass after exposure.

**Figure 8:**
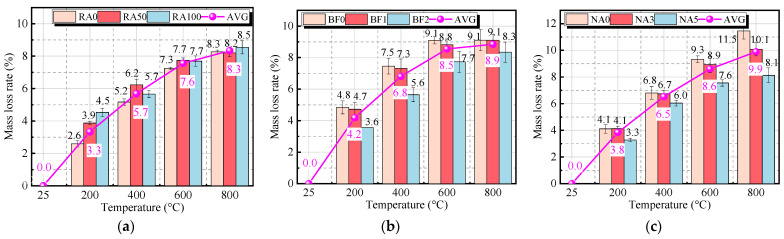
Mass loss rate of specimens after high temperature: (**a**) RA group (r_RA_ = 0/50/100%); (**b**) BF group (r_BF_ = 0/1/2%); (**c**) r_NA_ group (rNA = 0/3/5%).

Figure 8 shows the mass loss rate of the concrete specimens after high-temperature exposure. The mass loss rate of BF and RA-containing concrete increases significantly with temperature. Below 200 °C, the mass loss primarily results from the evaporation of free water. Between 200 °C and 400 °C, the mass loss rate accelerates, mainly due to the decomposition of C-S-H gel and the evaporation of gel water. As the temperature rises to 400–600 °C, the evaporation of free water and gel water is nearly complete, and mass loss is primarily due to the decomposition of Ca(OH)_2_, with a slower rate of increase. Above 700 °C, the mass loss rate increases primarily due to the decomposition of CaCO_3_ [40], and at 800 °C, the mass loss rate reaches 8–9%. At the same temperature, concrete specimens with higher RA content exhibit a greater mass loss rate. This is due to the higher porosity and increased crack formation in specimens with high RA replacement, which provide more pathways for water vapor escape, leading to more significant mass loss.

Furthermore, specimens with BF and NA exhibit lower mass loss rates than conventional UHPC-RA at high temperatures. At 200 °C, the mass loss rate is 3.3–4.8%. Between 200 °C and 400 °C, the mass loss rate increases significantly, mainly due to the loss of gel water and the decomposition of silicon hydroxide (H_4_SiO_4_). At 600 °C, the mass loss rate is 7.6–9.3%, and at 800 °C, it reaches 8.1–11.5%, primarily due to the decomposition of C-S-H gel and calcium carbonate. Overall, the incorporation of BF effectively slows the mass loss of concrete at high temperatures, while NA particles enhance its thermal stability. As shown in Figure 3, the XRD patterns indicate that NA exhibits a stable α-phase, demonstrating high melting points and good thermal stability.

### 3.2. Compressive Strength at High Temperatures

Table 7 presents the mechanical properties of the UHPC-RA specimen group with different mix proportions, including compressive strength, splitting tensile strength, and elastic modulus, to assess the influence of various factors on performance.

**Figure 9:**
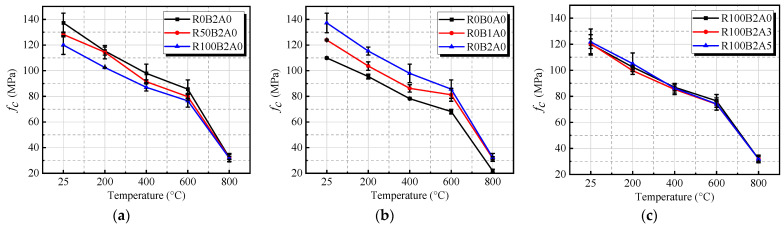

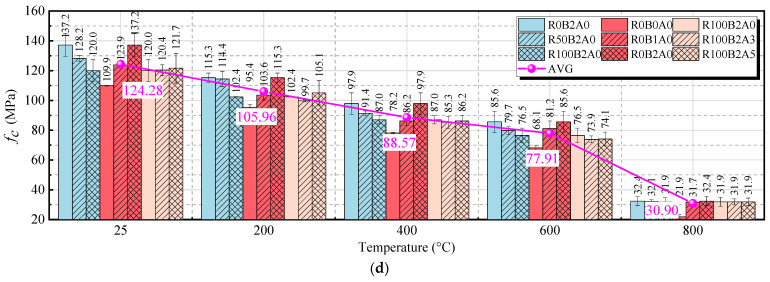
Experimental results on compressive strength with different temperatures: (**a**) effect of the r_RA_; (**b**) effect of the r_BF_; (**c**) effect of the r_NA_; (**d**) overall variation trend of compressive strength.

Figure 9 illustrates the variation in compressive strength (*f_c_*) of UHPC-RA specimens with different RA, BF, and NA contents as temperature increases. Overall, the compressive strength of concrete decreases with rising temperature, particularly between 600 °C and 800 °C, where the reduction is more pronounced. From ambient temperature to 200 °C, the compressive strength decreases slightly, ranging from 10.6% to 17.9%. Between 200 °C and 400 °C, the decrease is more substantial, ranging from 17.5% to 30.7%. This behavior is attributed to the evaporation of some moisture, which leads to strength reduction. As the temperature exceeds 400 °C, high temperatures significantly impact compressive strength, with reductions of 34.5–39.1% at 600 °C and 73.4–80.1% at 800 °C, mainly due to the decomposition of C-S-H gel, Ca(OH)_2_, and dehydration of calcium carbonate. Furthermore, the incorporation of BF significantly influences the high-temperature compressive strength of the concrete.

Figure 9a shows the compressive strength variation with temperature for specimens with different RA replacement ratios (0%, 50%, 100%), which generally follow the same downward trend. The 0% RA replacement specimen (R0B2A0) exhibits compressive strength at 600 °C and 800 °C as 37.6% and 71.4% of its value at ambient temperature, respectively, while the 100% RA replacement specimen (R100B2A0) shows compressive strengths of 36.3% and 83.4% at 600 °C and 800 °C, respectively. At 800 °C, all three specimens experience a significant drop in compressive strength, with higher RA replacement ratios leading to a more pronounced reduction. The greater the temperature exposure, the lower the residual strength, as cracks form on the specimen’s surface and interior, especially in the 100% RA replacement specimen, where the increased porosity disrupts the internal cementitious structure and deteriorates the material performance. Figure 9b shows that UHPC-RA with 1 kg/m^3^ and 2 kg/m^3^ BF content consistently exhibits superior compressive strength at all temperatures compared to conventional UHPC-RA. In particular, at 800 °C, the compressive strength increases by 44.8–47.9%, and at 600 °C, the increase ranges from 19.2% to 25.7%. However, when the BF content increases to 2 kg/m^3^, the compressive strength improvement becomes more significant. It is important to note that excessive fiber content may lead to fiber clumping during mixing, resulting in poorer high-temperature performance. Further analysis reveals that Figure 9c shows that the incorporation of NA particles does not significantly improve compressive strength. After temperature exposure at 20 °C, 200 °C, 400 °C, 600 °C, and 800 °C, the compressive strength of UHPC-RA with 5% NA content (R100B2A5) is improved by 1.42%, 2.64%, 0.92%, 3.14%, and 0.00%, respectively, compared to the specimen without NA content (R100B2A0). In conclusion, the compressive strength of BF-modified UHPC-RA concrete under high-temperature conditions exhibits a complex variation, influenced by various factors, including temperature, fiber content, and nano-material incorporation.

### 3.3. Splitting Tensile Strength at High Temperatures

**Figure 10:**
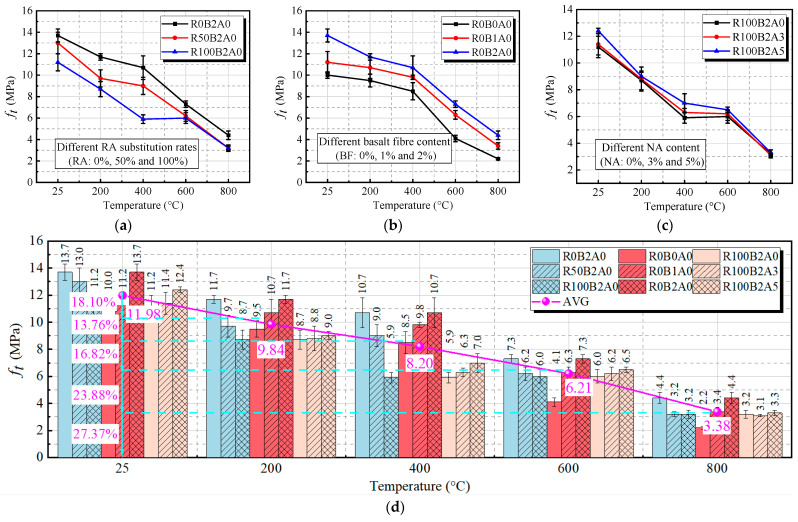
Experimental results on splitting tensile strength with different temperatures: (**a**) effect of the r_RA_; (**b**) effect of the r_BF_; (**c**) effect of the r_NA_; (**d**) overall variation trend of splitting tensile strength.

At high temperatures, the splitting tensile strength (*f_t_*) of BF and RA blended UHPC-RA concrete is crucial for evaluating its tensile performance and crack formation under extreme conditions. The experimental results shown in Figure 10 indicate that the splitting tensile strength decreases with increasing temperature, regardless of RA replacement ratio and BF content. From 25 °C to 800 °C, the splitting tensile strength decreases sharply. For example, at 25 °C, the splitting tensile strength of R0B2A0 is 13.7 MPa, but at 800 °C, it drops to 3.4 MPa. This overall trend suggests that higher temperatures lead to deterioration of the internal structure, likely due to moisture release and expansion of aggregates [40], which results in increased cracks and reduced tensile strength.

Figure 10a clearly shows that RA replacement has a significant effect on splitting tensile strength. As RA content increases, the strength decreases, particularly at higher replacement rates (50% and 100%). At 25 °C, the splitting tensile strength is highest with 0% RA replacement (13.7 MPa), and decreases progressively with higher RA content, reaching 11.2 MPa at 100% replacement. This is due to RA inducing more porous structures, which weaken tensile strength. As shown in Figure 10b, the incorporation of BF improves the splitting tensile strength of UHPC-RA. After exposure to temperatures of 20 °C, 200 °C, 400 °C, 600 °C, and 800 °C, the splitting tensile strength of UHPC-RA with 2 kg/m^3^ BF content is increased by 37.0%, 23.2%, 25.9%, and 78.0%, compared to the specimen without BF. Notably, at 800 °C, the BF-containing specimen (R0B2A0) shows a 100% increase in strength compared to the specimen without BF (R0B0A0). This suggests that BF helps mitigate the high-temperature damage that typically weakens material strength, likely due to BF’s ability to effectively leverage its tensile strength to prevent rapid separation of concrete aggregates and the cement matrix during high-temperature tensile failure. Furthermore, Figure 10c shows that the NA content has some positive influence on the splitting tensile strength of UHPC-RA, although the overall improvement is between 3.4% and 19.3%. After the addition of 5% NA, the splitting tensile strength of UHPC-RA (R100B2A5) increases by 10.98%, 3.68%, 19.32%, 8.17%, and 3.44%, respectively, compared to the specimen without NA (R100B2A0). NA incorporation improves the splitting tensile strength of UHPC-RA, particularly at 400 °C, where the splitting tensile strength of nanoalumina-modified UHPC-RA improves from 5.9 MPa to 7.0 MPa.

In summary, the inclusion of BF and optimal NA content can significantly enhance the high-temperature tensile performance of UHPC-RA concrete. BF plays a critical role in maintaining structural integrity at high temperatures. The combination of BF and NA in UHPC-RA strengthens the material’s resistance to thermal damage, making it a promising candidate for high-temperature applications.

**Figure 11:**
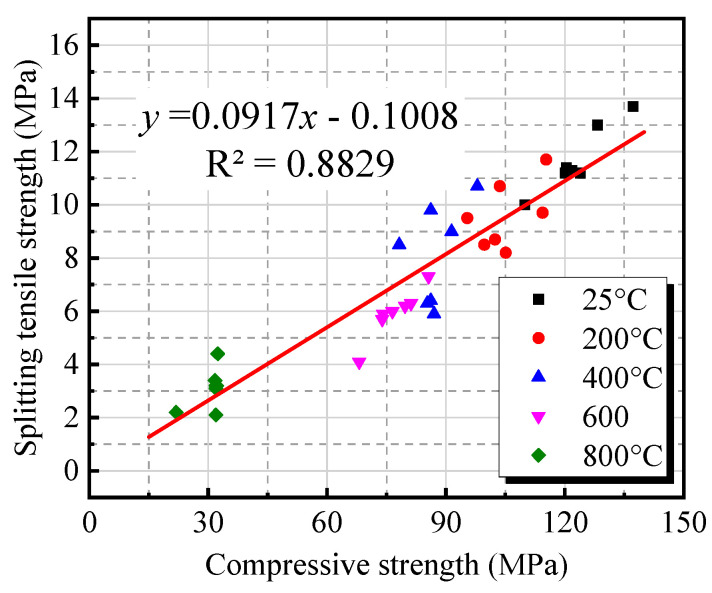
The relationship between compressive and splitting tensile strength of concrete at different temperatures.

Figure 11 illustrates the relationship between compressive strength and splitting tensile strength of BF-modified UHPC-RA concrete at different temperatures. The data show that both compressive strength and splitting tensile strength decrease as the temperature increases. Specifically, the relationship between the two remains linear, with a fitting coefficient of R^2^ = 0.8829, indicating a strong correlation, which is consistent with the findings of Silva et al. [46], Mefteh et al. [47], and Dilbas et al. [48]. This suggests that, under high-temperature conditions, although the compressive strength of concrete significantly decreases, the reduction in splitting tensile strength is considerably influenced, likely due to changes in the internal structure of the material.

### 3.4. Elastic Modulus

**Figure 12:**
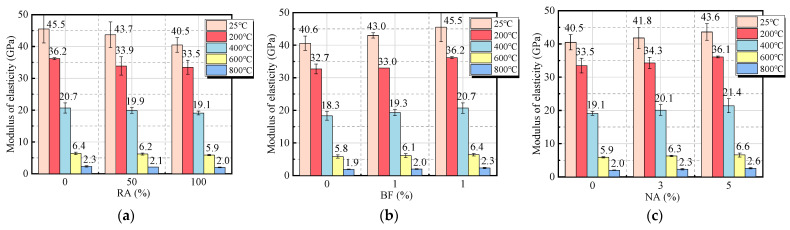

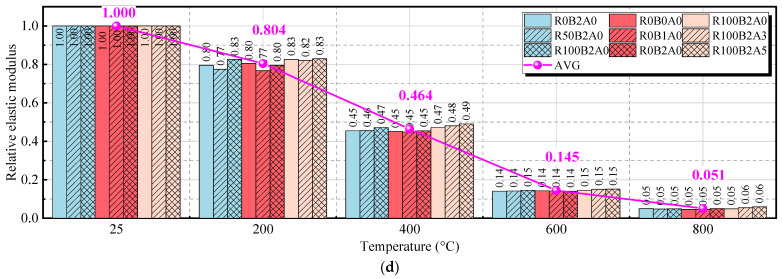
Comparison of elastic modulus of specimens with temperature: (**a**) effect of the r_RA_; (**b**) effect of the r_BF_; (**c**) effect of the r_NA_; (**d**) relative modulus.

Figure 12 presents the variation in the elastic modulus of BF-modified UHPC-RA concrete under different temperature conditions, highlighting the significant impact of high temperature on its mechanical properties. The experimental results indicate that the elastic modulus of the concrete decreases progressively with temperature, with a more noticeable reduction below 400 °C. In the temperature range of 600–800 °C, the reduction rate becomes more gradual. This phenomenon is mainly attributed to the evaporation of moisture and the thermal expansion of aggregates, which increase internal porosity and reduce the concrete’s density, thereby weakening its resistance to deformation [42]. Specifically, at ambient temperature, the elastic modulus of R0B2A0 is 45.5 GPa, which decreases to 36.2 GPa at 200 °C, further drops to 20.7 GPa at 400 °C, becomes 6.4 GPa at 600 °C, and reaches only 2.3 GPa at 800 °C. This suggests that as the temperature increases, the concrete’s ability to deform increases significantly, with the substantial decline in elastic modulus closely related to the degree of internal structural damage.

Figure 12a indicates that the incorporation of RA has a negative impact on the elastic modulus of UHPC-RA. Experimental results show that at ambient temperature, the specimen with 100% RA replacement (R0B2A0) exhibits the highest elastic modulus, which is 3.96% and 10.99% higher compared to specimens with lower RA content (R50B2A0 and R100B2A0). At temperatures of 200 °C, 400 °C, 600 °C, and 800 °C, the addition of RA accelerates the decline in elastic modulus, especially at higher temperatures, where the modulus stabilizes at a relatively low level. This is because RA typically has lower density and higher porosity, making the concrete more susceptible to damage at elevated temperatures.

Figure 12b demonstrates that the incorporation of BF has a positive effect on the elastic modulus of concrete. When the BF content reaches 2 kg/m^3^, the static elastic modulus of the specimen reaches its highest value, with an increase of 6% to 19% compared to the specimen without BF. This shows that BF can effectively improve the concrete’s resistance to deformation at high temperatures. At elevated temperatures, BF incorporation helps slow the decline in the elastic modulus, especially in the low to medium temperature range, where the modulus retention is more significant.

However, the effect of NA content on the high-temperature performance of UHPC-RA is limited, as shown in Figure 12c. At temperatures above 600 °C, the positive impact diminishes, despite the high thermal stability of NA particles playing an important role. At elevated temperatures, the cement matrix becomes more porous, preventing the material from exhibiting its full mechanical properties.

Figure 12d shows the relative change in the elastic modulus of each specimen. In general, as the temperature increases, the relative elastic modulus of all materials decreases. At 25 °C, the relative elastic modulus of all materials is close to 1.0. As the temperature rises to 200 °C, the average relative elastic modulus decreases to 0.804. At 400 °C, it further decreases to 0.464, and at 800 °C, the values for all materials drop below 0.1. Although the incorporation of BF can improve the elastic modulus of UHPC-RA concrete and slow down the impact of high temperatures on its deformation ability, the increase in temperature still causes significant damage to the elastic modulus of the concrete, especially when the temperature exceeds 600 °C. Therefore, optimizing the BF content and reasonably controlling the RA replacement rate are crucial for enhancing the high-temperature durability of concrete.

### 3.5. Comprehensive Evaluation of Mechanical Performance

The previously discussed mechanical indicators evaluate individual performance and cannot provide a holistic assessment of the material’s overall properties. Here, we refer to the concept of the harmonic mean to define a comprehensive mechanical performance parameter, *CP*. This parameter utilizes the set of mechanical indicators to describe the material’s overall performance under a specific mix, considering the influence of the minimum value in the data set. The harmonic mean accounts for the limiting effects of the weakest property, providing an evaluation of the material’s performance based on the “bottle neck” effect. Thus, the comprehensive mechanical performance parameter CP is shown in Equation (5):

(5) $$CP=\frac{3}{w_{c}\frac{1}{f_{c}}+w_{t}\frac{1}{f_{t}}+w_{E}\frac{1}{E}}$$

To calculate *CP*, we select three typical mechanical indicators—compressive strength (*f_c_*), splitting tensile strength (*f_t_*), and elastic modulus (*E*)—and normalize these indicators to account for differences in their magnitudes. Here, *w_c_*, *w_t_*, and *w_E_* represent the weight coefficients for compressive strength, splitting tensile strength, and elastic modulus, respectively. These weight coefficients are defined as the inverse of the average value of each corresponding indicator (i.e., $w_{c}=1/\bar{f_{c}}$, and similarly for *w_t_* and *w_E_*). For other mechanical indicators, the selection of indicators should meet the following criteria: they should represent different mechanical properties, have distinct and independent characteristics, and, when indicators are not at the same level, the weight coefficients (*w*) should be adjusted accordingly. The calculated comprehensive index should be compared with the control group values. For comparison purposes, the relative comprehensive performance (*RCP*) is defined as the ratio of the comprehensive mechanical performance parameter *CP* of the specimen to that of the control specimen (R0B0A0).

(6) $$RCP=\frac{CP}{CP_{Control\;specimen}}$$

**Figure 13:**
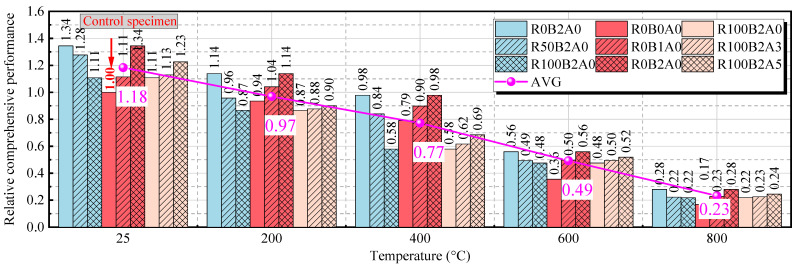
Comparison of relative comprehensive performance (*RCP*) of different specimens at different temperatures.

Figure 13 presents the relative comprehensive mechanical performance (*RCP*) of BF-modified UHPC-RA concrete at different temperatures. As shown, compared to the control specimen (R0B0A0), the figure enables a clear comparison of the effect of different mix proportions on the material’s overall performance enhancement. Furthermore, as temperature increases, the overall mechanical performance of the concrete decreases. At 25 °C, all specimens show relatively high *RCP* values, with the R0B2A0 specimen performing the best at 1.34, and the average *RCP* value is 1.18. As the temperature rises to 200 °C, the *RCP* value begins to decrease, with an average value of 0.97, indicating significant impact on the mechanical properties of the concrete. With a further temperature increase to 400 °C, the *RCP* value continues to drop, with an average of 0.77, demonstrating a further weakening of the mechanical properties. At 600 °C, the *RCP* value significantly decreases to 0.49, indicating a substantial decline in mechanical performance at high temperatures. At 800 °C, the *RCP* value reaches its lowest point, with an average of 0.23, showing severe degradation of the material’s mechanical properties under extreme high temperatures.

These results indicate that the mechanical properties of BF-modified UHPC-RA concrete are significantly reduced under high-temperature conditions, with performance degradation becoming more pronounced as the temperature increases. This trend is crucial for evaluating and designing concrete structures in high-temperature environments, highlighting the importance of material selection and structural design to ensure the safety and reliability of structures in such conditions.

### 3.6. DIC Measurement Results

**Figure 14:**
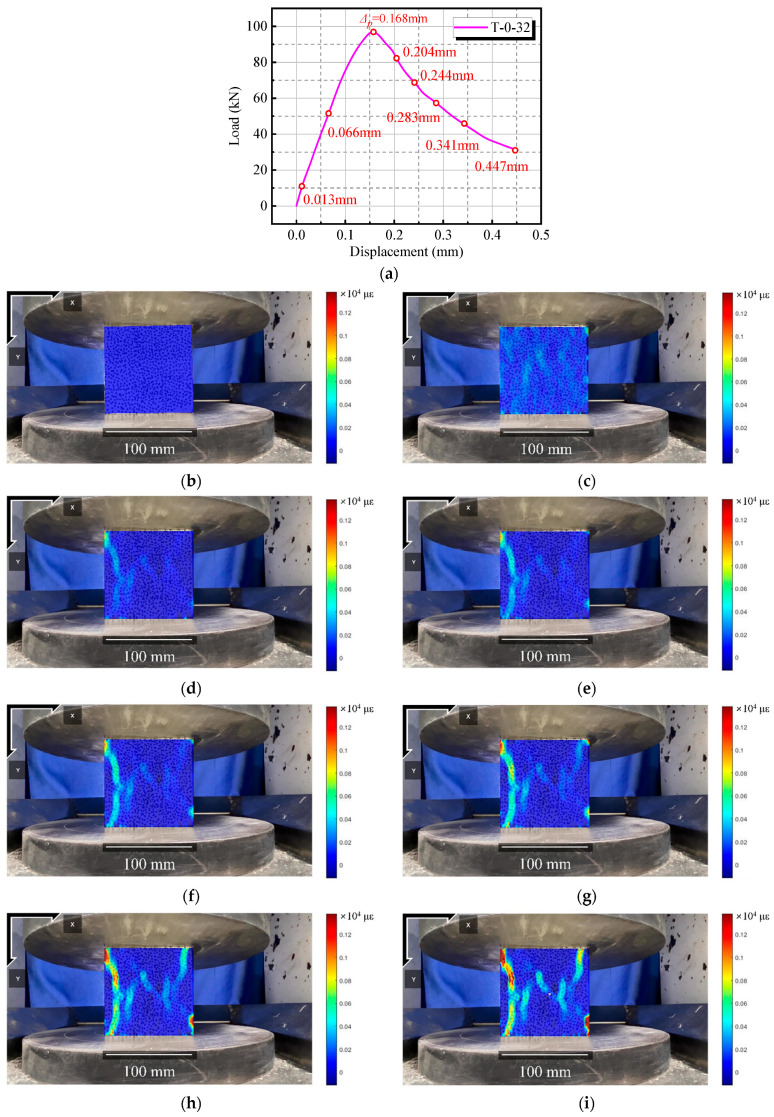
DIC measurement of crack evolution in compression test (R100B2A5 specimen) at room temperature: (**a**) load–displacement curve; (**b**) 0.013 mm; (**c**) 0.066 mm; (**d**) 0.168 mm; (**e**) 0.204 mm; (**f**) 0.244 mm; (**g**) 0.283 mm; (**h**) 0.341 mm; (**i**) 0.447 mm.

Figure 14 shows the DIC measurement results for UHPC-RA concrete under room temperature conditions, utilizing the DIC monitoring system outlined in Section 2.7 to calculate the changes in image data during the specimen loading process, revealing crack evolution and strain distribution during compression testing. Figure 14a show the load–displacement curve for the R100B2A5 specimen at room temperature, with characteristic points corresponding to different displacement stages. In the initial stage (Figure 14b,c), no noticeable cracks appear on the specimen surface, and the strain distribution is uniform and small, though slight changes in the surface strain field can be detected by DIC. As the vertical displacement increases to 0.168 mm and 0.204 mm (Figure 14d,e), the DIC results show the formation of strain concentration regions, indicating the initiation of microcracks. When the vertical displacement reaches 0.244 mm and 0.283 mm (Figure 14f,g), the main cracks begin to expand, and the strain concentration region significantly increases, indicating further crack development. At vertical displacements of 0.341 mm and 0.447 mm (Figure 14h,i), the specimen enters the final failure stage, with the main crack passing through the specimen as shown by the red regions.

**Figure 15:**
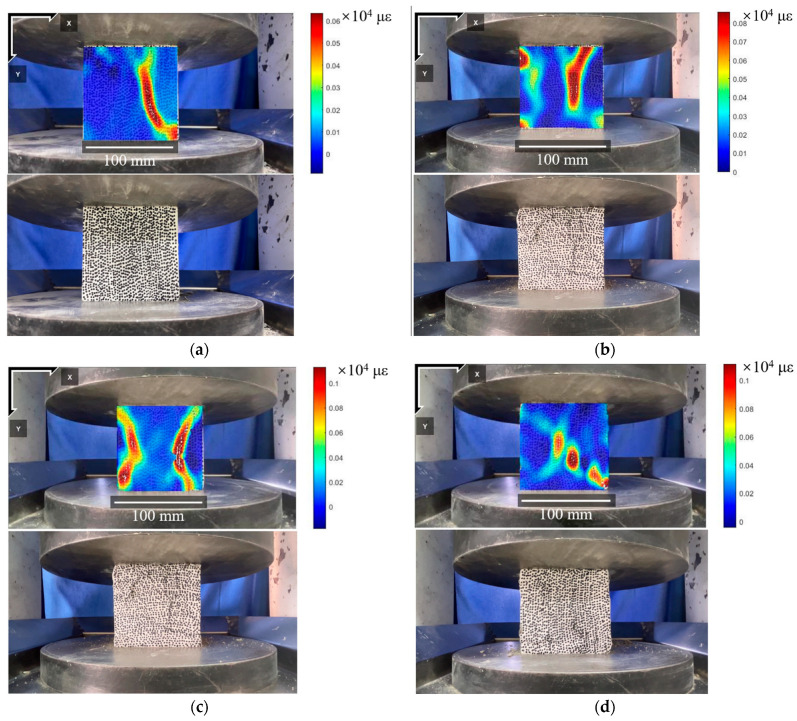
DIC measurement results of R100B2A5 specimen failure at different temperatures: (**a**) 200 °C; (**b**) 400 °C; (**c**) 600 °C; (**d**) 800 °C.

Figure 15 shows the final failure morphology of the R100B2A5 specimen under different high-temperature conditions. At 200 °C (Figure 15a), crack propagation is more pronounced, with increased strain concentration near the primary cracks. As the temperature rises to 400 °C (Figure 15b), crack expansion becomes more severe, with the strain concentration area further increasing and multiple primary cracks appearing. At 600 °C (Figure 15c) and 800 °C (Figure 15d), the specimen experiences more extensive crack propagation, with significantly increased strain concentration areas and failure modes exhibiting more primary cracks. After failure, the remaining specimen is no longer intact, indicating that material damage increases under high-temperature conditions.

Comparing Figure 14 and Figure 15, it is evident that high temperatures significantly affect crack propagation and strain distribution in UHPC-RA concrete. At room temperature, crack propagation is slower, and the strain concentration areas are smaller. However, under high-temperature conditions, crack propagation accelerates, and the strain concentration areas expand significantly, demonstrating that high temperatures exacerbate material damage. Additionally, high temperatures cause an increase in specimen deformation. In summary, DIC measurements reveal that the crack propagation and strain distribution of UHPC-RA concrete under high-temperature conditions differ significantly from those at room temperature. High temperatures accelerate crack formation and propagation, enlarging strain concentration areas and intensifying material damage.

### 3.7. SEM Analysis

**Figure 16:**
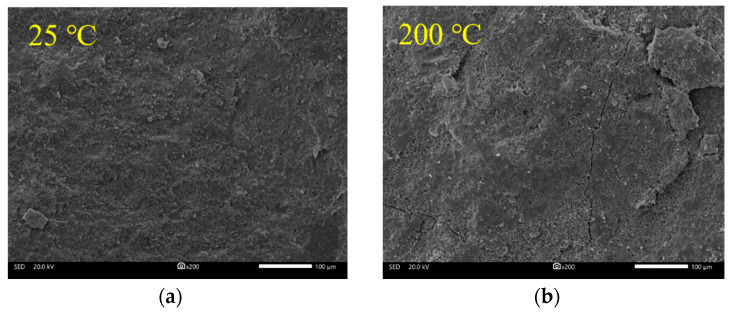

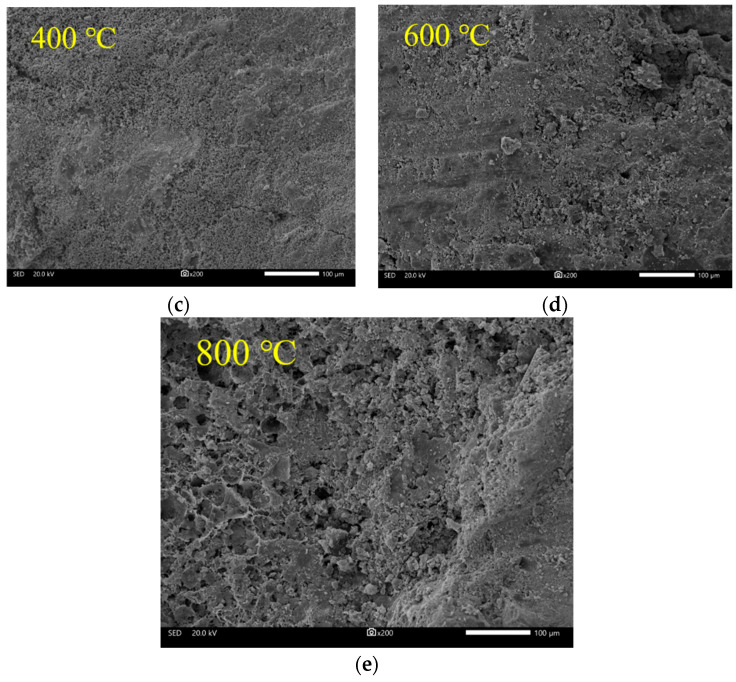
SEM images of the sample mortar matrix with different temperatures: (**a**) 25 °C; (**b**) 200 °C; (**c**) 400 °C; (**d**) 600 °C; (**e**) 800 °C.

**Figure 17:**
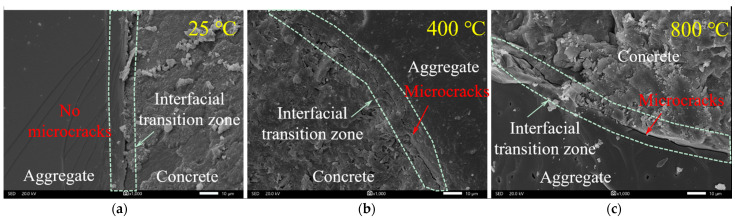
SEM images of the interfacial transition zone for R50B2A0 specimen with different temperatures: (**a**) 25 °C; (**b**) 400 °C; (**c**) 800 °C.

**Figure 18:**
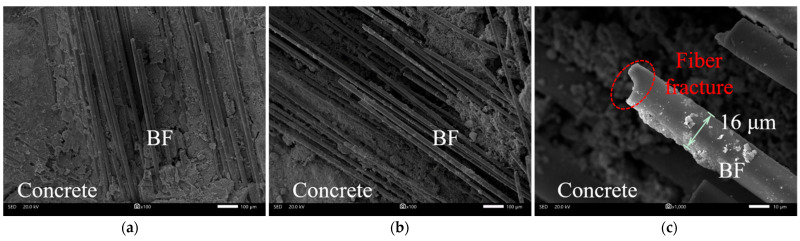
SEM images of basalt fibers with different BF contents: (**a**) 1 kg/m^3^; (**b**) 2 kg/m^3^; (**c**) fracture surface of pulled BFs.

**Figure 19:**
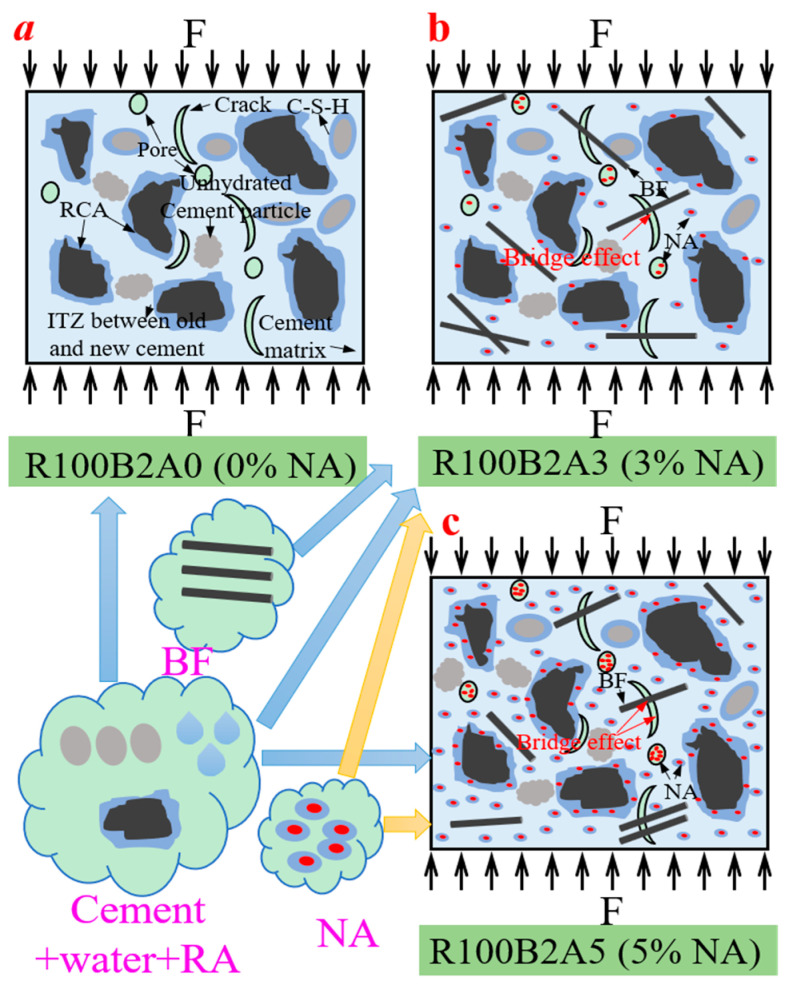
Schematic diagram of the cementitious system with different fiber ratios and NA contents: (**a**) R100B2A0 (0% NA); (**b**) R100B2A3 (3% NA); (**c**) R100B2A5 (5% NA).

To more intuitively analyze the changes in the internal microstructure of UHPC-RA specimens at high temperatures, SEM was used to examine the cement matrix, aggregate-cement matrix interfacial transition zone (ITZ), and fiber–cement matrix morphology at different temperatures. The experimental results are shown in Figure 16, Figure 17, Figure 18 and Figure 19.

(1) Microstructure of the Cement Matrix

The SEM images in Figure 16 reveal the evolution of the cement matrix microstructure of UHPC-RA concrete under various temperature conditions. At room temperature (25 °C, Figure 16a), the cement matrix exhibits a dense structure, with hydration products such as C-S-H gel and Ca(OH)_2_ crystals intact, forming a continuous and regular structure with minimal pores and cracks. This indicates sufficient cement hydration, providing the matrix with high density and strength. As the temperature increases to 200 °C (Figure 16b), slight loosening of the cement matrix occurs, with the loss of free water and some crystallization water leading to the formation of microcracks and pores. However, the C-S-H gel structure remains intact, and cement hydration continues, resulting in a partial increase in compressive strength. At 400 °C (Figure 16c), free water and capillary pore water in the cement matrix are mostly evaporated, and some adsorbed and interlayer water in C-S-H gel begins to evaporate, weakening the matrix’s structural bonds, increasing porosity, and causing internal microcracks. At this point, ettringite (AFt) crystals are completely decomposed by heat, and Ca(OH)_2_ begins to decompose slightly, though the aggregate structure remains unchanged. As the temperature rises to 600 °C (Figure 16d), the cement matrix becomes more porous, with most hydration products decomposed, Ca(OH)_2_ undergoing substantial dehydration and losing its original form. The bond strength between hydrated and unhydrated particles weakens, and the cement matrix begins to fragment, with the C-S-H gel network structure damaged, resulting in a loose structure and the formation of crystalline clusters with volume shrinkage. At this stage, the UHPC-RA structure is severely damaged, as a large amount of Ca(OH)_2_, after dehydration, turns into free CaO, which rehydrates upon cooling, causing volume expansion and significant internal stress, leading to internal expansion and a substantial decrease in density. Finally, at 800 °C (Figure 16e), the cement matrix’s density further decreases, with an increase in pore volume and extensive cracking, resulting in severe damage to the matrix and the near-complete disappearance of crystalline products. This is due to the complete dehydration of C-S-H gel, complete decomposition of Ca(OH)_2_, and decomposition of CaCO_3_ into CaO [39]. Macroscopically, the compressive strength of the specimen decreases significantly, with a residual strength ratio of only 30–40%.

In summary, as the temperature increases, the cement matrix of UHPC-RA concrete transforms from a dense to a loose structure, with increasing porosity and cracks, leading to the decomposition of hydration products and a significant reduction in mechanical performance. This process involves both the physicochemical changes of cement hydration products and the internal stresses and volume changes induced by temperature. These phenomena are closely related to the macroscopic mechanical performance changes observed in the UHPC-RA specimens.

(2) Cement and Additives

The SEM images in Figure 17 provide a detailed view of the microstructural changes in the ITZ between the aggregate and cement paste of the R50B2A0 sample at 25 °C, 400 °C, and 800 °C. At 25 °C (Figure 17a), the ITZ is dense, with no microcracks, indicating a good bond between the cement paste and the aggregate, which is crucial for the overall mechanical performance of the concrete. As the temperature increases to 400 °C (Figure 17b), microcracks begin to appear in the ITZ, caused by inconsistent thermal expansion between the aggregate and matrix, as well as shrinkage of the paste due to dehydration of hydration products. These factors collectively cause the bond strength in the ITZ to deteriorate. At 800 °C (Figure 17c), microcracks in the ITZ increase significantly, and the structure is more severely damaged, indicating that high temperatures further promote the separation between the cement paste and aggregate, significantly weakening the bond strength of the interface. These observations reveal the transformation of the ITZ structure from intact to cracked with increasing temperature, with the increase and expansion of microcracks notably reducing the bond strength [42]. This change in interface structure directly affects the macroscopic mechanical performance of concrete, especially in high-temperature environments. Therefore, a deep understanding and control of the ITZ microstructural evolution are critical to enhancing the performance of UHPC-RA concrete at high temperatures.

(3) Cement and Additives

Figure 18 displays the SEM images of the fiber distribution in UHPC-RA concrete with different BF contents, showing the fiber distribution at 1 kg/m^3^ and 2 kg/m^3^ BF content, as well as the fracture surface of the BFs after pull-out. These images allow for the observation of the distribution of BFs in the concrete matrix and their interface bonding with the matrix. The interaction between fibers at different contents shows significant microstructural differences. SEM images reveal that at lower fiber contents (1 kg/m^3^, Figure 18a), the fibers are embedded in the matrix with relatively uniform hydration products surrounding them. This strong bond between the fibers and the matrix leads to an effective fiber–matrix interface with minimal cracking. However, as the fiber content increases (2 kg/m^3^, Figure 18b), the interface appears slightly disturbed, likely due to increased competition for bonding sites between fibers and the matrix, potentially weakening the overall bonding strength. Additionally, at high temperatures (Figure 18c), visible fiber fracture occurs on the surface, typically around 16 µm, indicating mechanical failure due to thermal stress, which significantly reduces the composite material’s mechanical performance.

In UHPC-RA concrete, the incorporation of BFs helps to transfer and coordinate internal forces within the concrete structure under stress, thereby improving stress distribution and delaying the appearance of brittle regions, thus enhancing the concrete’s mechanical strength. The addition of fibers acts as a bridging agent, reinforcing the overall integrity of the concrete. Due to the high specific surface area of BFs, their bond strength with the matrix is stronger, which enhances the concrete’s toughness and crack resistance. On the microstructural level, the ITZ between BFs and the cement matrix plays a crucial role in fiber reinforcement. A strong interface bond ensures that the fibers can effectively enhance the concrete. However, high-temperature environments may impact the bond strength between the fibers and the matrix, causing significant damage to the ITZ, including microcracks and fiber buckling, further exacerbating the loss of bond strength and reducing mechanical performance [6]. For example, at 800 °C, BFs may undergo oxidation and degradation, leading to a decrease in bonding strength, thus affecting the fiber bridging effect and its role in the concrete.

### 3.8. Mechanism Analysis

Figure 19 presents a schematic of the cement matrix system in UHPC-RA concrete with different BF ratios and NA contents. This figure illustrates the mechanism by which fibers influence the microstructure and mechanical properties of the concrete at different NA contents. In the R100B0A0 specimen, where no NA is added, the lack of the densification effect from nano-materials results in numerous pores inside the concrete, leading to relatively low mechanical properties. However, in the R100B2A3 specimen, with 3% NA, the NA particles promote cement hydration due to their high surface area, forming more C-S-H gel while filling pores and the ITZ, thereby improving the concrete’s density and strength. The addition of NA promotes the formation of C-S-H gel through chemical reactions, while consuming Ca(OH)_2_, which negatively impacts strength, further improving strength. The chemical reaction equation is shown in Equation (7):

(7) $$Al_{2}O_{3}+mH_{2}O+nCa{\left(OH\right)}_{2}\to nCaO\cdot Al_{2}O_{3}\cdot (m+n)H_{2}O$$

As shown in Figure 19a, after adding RA, NA fills pores and the ITZ between new and old cement, resulting in a denser microstructure, reducing porosity, and enhancing the cohesion of the cementitious system. The high surface area of NA accelerates the cement hydration reaction, generating more hydration products, further strengthening the concrete. When fibers combine with NA, the bond between fibers and the cement matrix is effectively enhanced, reducing stress concentration at crack tips and controlling crack propagation. For example, in the R100B2A3 specimen, BFF fibers play an active role in bridging the NA-modified cement matrix (Figure 19b), while in the R100B2A5 specimen, the synergistic use of NA and fibers reinforces the UHPC-RA e concrete (Figure 19c). This synergistic effect is achieved through the strengthening role of nano-materials at the microscopic scale and the reinforcement of fibers at both macro and micro scales.

Furthermore, when NA and fibers are used together, their coupling effect promotes the bonding between fibers and the matrix, effectively reducing stress concentration at surface defects. For instance, in the R100B2A5 specimen with 5% NA, the combined effect of mixed fibers through the strengthening action of NA nano-materials at the microscopic scale and the reinforcing role of fibers at the macroscopic scale significantly improves the compressive strength, splitting tensile strength, and elastic modulus of the concrete. BFs form a three-dimensional random network structure inside the concrete, acting as “secondary reinforcement,” which, especially in high-temperature environments, can limit crack propagation due to their high melting point, thereby maintaining relatively high residual strength. In conclusion, through reasonable design of fiber ratios and NA content, the mechanical and high-temperature resistance properties of UHPC-RA concrete can be effectively improved.

## 4. Conclusions

### 4.1. Research Conclusions

This study investigated the mechanical properties and microstructure of BF and NA-modified UHPC-RA under high-temperature conditions. The following conclusions can be drawn from the results:

1. Under high-temperature exposure, UHPC-RA concrete-containing BF and RA exhibited significant changes in appearance. As the temperature increased, the surface color of the specimens gradually lightened, cracks increased, and peeling occurred, especially at 800 °C, where cracks became more prominent. The incorporation of BF effectively suppressed crack propagation, particularly at higher temperatures. Specimens with a higher BF content demonstrated better crack resistance, with the initial cracking temperature being lower, thereby enhancing the high-temperature integrity of the concrete. Additionally, the mass loss rate significantly increased under high temperatures, but the incorporation of BF and NA effectively slowed mass loss, showing better thermal stability.
2. The compressive strength, splitting tensile strength, and elastic modulus of the specimens all showed a decreasing trend as the temperature increased. Notably, compressive strength decreased significantly above 600 °C. However, the inclusion of BF effectively enhanced the compressive strength at high temperatures. At 800 °C, the compressive strength increased by 44.8% to 47.9% compared to specimens without BF. While NA particles had a limited effect on compressive strength improvement, they contributed to the enhancement of splitting tensile strength, particularly at 400 °C, where they significantly increased the splitting tensile strength of the concrete.
3. The relative comprehensive performance (RCP) parameter was used to quantify the effect of different mix ratios on the overall performance of UHPC-RA and the temperature-induced decline in mechanical properties. The results indicated that as the temperature increased, the comprehensive mechanical performance of the specimens gradually decreased, with the RCP value reaching its lowest at 800 °C, indicating that high temperature had a significant impact on the mechanical properties of the concrete. Proper amounts of BF and NA could effectively improve the material’s overall performance, with the synergistic effect of BF and NA significantly improving the mechanical properties of concrete under high-temperature conditions.
4. DIC technology was used to monitor strain and crack development in concrete during loading. The results showed that at room temperature, strain distribution was uniform, and crack propagation was slow. However, at high temperatures, crack propagation accelerated, and strain concentration areas became significantly larger, indicating that high temperature aggravated concrete damage. At 800 °C, cracks penetrated the entire specimen, with severe damage.
5. SEM analysis revealed that with increasing temperature, the cementitious matrix transitioned from a dense structure to a porous one, with an increase in porosity and decomposition of hydration products, leading to a decline in mechanical properties. The bridging effect of BFs significantly enhanced the crack resistance of concrete, particularly at high temperatures, where BFs effectively limited crack propagation and reduced high-temperature damage. NA particles improved the high-temperature resistance of concrete by filling voids and enhancing the densification of the interfacial transition zone (ITZ), with the synergistic effect of NA and BF significantly improving the mechanical properties and thermal stability of the material.

From an industry perspective, this study provides new choices for concrete materials in high-temperature environments for the construction industry, especially in the event of a fire, which is of great significance for the application of high-rise buildings, bridges, tunnels, and other structures. By adopting UHPC-RA with good high-temperature stability, the disaster resistance of the structure can be significantly improved under extreme conditions such as fire. At the same time, the use of recycled aggregates (RAs) can not only improve the sustainability of materials, but also promote the development of green building technology and help with environmental protection and resource recycling. For research purposes, this study extends the theoretical basis for the behavior of UHPC under high-temperature conditions and provides experimental data and methodological support for future related research. In the future, scholars can further explore the effects of more mineral admixtures, fiber materials, and nano additives on the high-temperature performance of concrete, promoting the application and development of concrete materials in extreme environments. In summary, the results of this study provide theoretical support and practical guidance for the application of UHPC in high-temperature environments, and have important industry application prospects.

### 4.2. Limitations and Suggestions for Future Research

Although this study evaluated the respective mechanical properties and SEM morphological microstructure of BF and NA particle-modified UHPC-RA under high-temperature conditions, it still has certain limitations. Firstly, this study primarily focused on the effects of specific temperature conditions on UHPC-RA, but it did not cover extreme temperatures (e.g., above 1000 °C) or longer exposure durations. Secondly, this study utilized specific types of RA and admixtures (e.g., BF and NA). Different sources of RA or other types of admixtures may have varying impacts on concrete performance, and the study did not consider a broader range of recycled aggregates or different admixture combinations to further assess their effects on high-temperature performance. Lastly, this study mainly employed conventional mechanical tests (e.g., compressive strength, tensile strength, elastic modulus tests), DIC testing, and SEM techniques to analyze the mechanical properties, failure evolution, and microstructure of the concrete. While these methods provided substantial data, they did not comprehensively cover all potential deep-seated physical mechanisms influencing high-temperature performance. The absence of more advanced non-destructive testing techniques (e.g., X-ray imaging, thermal analysis) may have limited a more in-depth understanding.

To address these issues, future research could focus on the following aspects:

1. The coupling mechanism between microstructure and mechanical properties could be further explored using advanced micro-characterization techniques such as X-ray micro-CT and SEM. This would allow for a deeper understanding of the interactions and synergistic effects of RA, BF, and NA particles at the micro-scale, and their collaborative repair mechanisms. Special attention should be given to the impact of these materials on the cement matrix, ITZ, and fiber–matrix interface transition zones under high-temperature conditions, revealing their specific mechanisms in improving mechanical properties.
2. Long-term performance evaluation under high-temperature exposure should be conducted. Future research should investigate the performance changes in UHPC-RA under prolonged high-temperature exposure, particularly the effects of long-term exposure on mechanical properties, durability, fatigue performance, and crack propagation behavior.
3. Research on the repair and modification of RAs should be undertaken to explore methods such as surface treatment, chemical modification, or the addition of functional fillers to improve the bond strength between RA and the cement matrix, and overall performance. In particular, modifying RA to improve its thermal stability and crack resistance under high-temperature conditions could enhance its performance in high-temperature environments.
4. Future studies could focus on the sustainability of UHPC-RA, assessing its environmental impact throughout the life cycle. Research should also investigate the balance between material cost and performance to achieve cost-effective mix designs, leading to more sustainable and economically viable building material solutions.

## Figures and Tables

**Table 1:** Physical properties of cementitious materials.

Properties	Physical Properties						Fineness (%)		
	Loss on Ignition	Specific Surface Area (cm^2^/g)	Specific Gravity	Moisture Content (%)	Water Absorption (%)	Mean Particle Size Diameter (μm)	Retained on 38 μm Sieve	Retained on 45 μm Sieve	Retained on 90 μm Sieve
Silica fume (SiF)	0.8	2250	2.25	0.15	5.6	24.56	15.6	12.13	3.54
Slag powder (SP)	0.7	4360	2.9	0.1	4.32	11.6	11.35	8.17	2.31
Portland cement (PC)	1.3	3580	3.1	0.08	2.3	15.3	8.65	5.04	0.78

**Table 3:** Physical properties of the basalt fibre (BF).

Length (mm)	Diameter (μm)	Density (kg/m^3^)	Modulus of Elasticity (GPa)	Number of Fibers per kg	Fracture Elongation (%)	Thermal Conductivity (W/m·K)	Melting Point (°C)	Tensile Strength (MPa)				
								25 °C	200 °C	400 °C	600 °C	800 °C
12	16	2700	85–110	~250 million	3	50.2	1450	3000–4800	2800–4500	2500–4000	2000–3500	1500–2500

**Table 4:** Physical properties of nanoalumina (NA) materials [38].

Product	Type	Crystal Form	Appearance	Particle Size D50 nm	Purity%	Specific Surface Area m^2^/g	Bulk Density g/cm^3^	PH Value	Viscosity (25 °C)	Place of Origin
Nanoalumina	TAP-A21	α-phase	White powder	30–40	>99.99	20	0.2	6~8	800cps	Nanjing

**Table 5:** UHPC-RA sample design and mixing ratio (kg/m^3^).

Mix No.	Tag	Water	SiF	SP	Sand	HPWR	Cement	NA	A	RA	BF
1	R0B2A0	220	224	206	550	6	430	0	1100	0	2
2	R50B2A0						430	0	550	550	2
3	R100B2A0						430	0	0	1100	2
4	R0B0A0						430	0	1100	0	0
5	R0B1A0						430	0	1100	0	1
6	R100B2A3						417.1	12.9	0	1100	2
7	R100B2A5						408.5	21.5	0	1100	2

Note: R = RA replacement rate, B = BF content and A = NA content. For example, R100B2A5 represents 100% RA replacement rate, 2 kg/m^3^ BF content, and 5% NA content.

**Table 6:** High-performance water-reducing (HPWR) agent performance parameters.

Physical property	Style	PH	Density (g/mL)	Water secretion rate (%)	Water reduction rate (%)	Viscosity (mPa·s)	Picture
	ZY-S type polycarboxylic acid	5.9	1.02	30	26	50	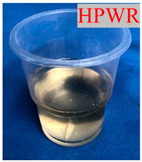
Effect on concrete	Initial setting time difference (min)	Final setting time difference (min)	Shrinkage ratio (MPa)	Effect on BF corrosion	Compressive strength ratio (%)		
					3 d	28 d	
	95	100	104	None	176	153	

**Table 7:** Mechanical properties after exposure to high temperatures.

Performance	Compressive Strength (MPa)					Splitting Tensile Strength (MPa)					Elastic Modulus (GPa)				
	25 °C	200 °C	400 °C	600 °C	800 °C	25 °C	200 °C	400 °C	600 °C	800 °C	25 °C	200 °C	400 °C	600 °C	800 °C
R0B2A0	137.2	115.3	97.9	85.6	32.4	13.7	11.7	10.7	7.3	4.4	45.5	36.2	20.7	6.4	2.3
R50B2A0	128.2	114.4	91.4	79.7	32.1	13	9.7	9	6.2	3.2	43.7	33.9	19.9	6.2	2.1
R100B2A0	120	102.4	87	76.5	31.9	11.2	8.7	5.9	6	3.2	40.5	33.5	19.1	5.9	2
R0B0A0	109.9	95.4	78.2	68.1	21.9	10	9.5	8.5	4.1	2.2	40.6	32.7	18.3	5.8	1.9
R0B1A0	123.9	103.6	86.2	81.2	31.7	11.2	10.7	9.8	6.3	3.4	43	33	19.3	6.1	2
R100B2A3	120.4	99.7	85.3	73.9	31.9	11.4	8.8	6.3	6.2	3.1	41.8	34.3	20.1	6.3	2.3

## Data Availability

The original contributions presented in this study are included in the article. Further inquiries can be directed to the corresponding author.

## Abbreviations

**Symbols**	**Content**
AE	Acoustic emission
BF	Basalt fiber
CP	Comprehensive mechanical performance
DIC	Digital image correlation
*E*	Elastic modulus
*fc*	Compressive strength
FRC	Fiber-reinforced concrete
*f* *_t_*	Splitting tensile strength
HPWR	High-performance water reducer
ITZ	Interfacial transition zone
NA	Nano alumina
NDE	Non-destructive evaluation
PC	Portland cement
RA	Recycled aggregates
RAC	Recycled aggregate concrete
RCP	Relative comprehensive performance
SCHSC	Self-compacting high-strength concrete
SCHSFRC	Self-compacting high-strength fiber-reinforced concrete
SEM	Scanning electron microscopy
SFRC	Steel fiber-reinforced concrete
SiF	Silica fume
SP	Slag powder
SSD	Saturated-surface-dry
UHPC	Ultra-high-performance concrete
UHPC-RA	Ultra-high-performance concrete with recycled aggregates
UPV	Ultrasonic pulse velocity
UTM	Universal testing machine
*wc*	Weight coefficient for compressive strength
*wE*	Weight coefficient for elastic modulus
*wt*	Weight coefficient for splitting tensile strength
XRD	X-ray diffraction

## References

[1] Feng C., Cui B., Huang Y., Guo H., Zhang W., Zhu J. (2022). Enhancement technologies of recycled aggregate–Enhancement mechanism, influencing factors, improvement effects, technical difficulties, life cycle assessment. Constr. Build. Mater..

[2] Liang C., Pan B., Ma Z., He Z., Duan Z. (2020). Utilization of CO_2_ curing to enhance the properties of recycled aggregate and prepared concrete: A review. Cem. Concr. Compos..

[3] Sollero M., Junior A.M., Costa C. (2021). Residual mechanical strength of concrete exposed to high temperatures–international standardization and influence of coarse aggregates. Constr. Build. Mater..

[4] Sun H., Luo L., Li X., Yuan H. (2024). The treated recycled aggregates effects on workability, mechanical properties and microstructure of ultra-high performance concrete Co-reinforced with nano-silica and steel fibers. J. Build. Eng..

[5] Sun H., Luo L., Yuan H., Li X. (2023). Experimental evaluation of mechanical properties and microstructure for recycled aggregate concrete collaboratively modified with nano-silica and mixed fibers. Constr. Build. Mater..

[6] Alaskar A., Albidah A., Alqarni A.S., Alyousef R., Mohammadhosseini H. (2021). Performance evaluation of high-strength concrete reinforced with basalt fibers exposed to elevated temperatures. J. Build. Eng..

[7] Luo L., Shi J., Wang J., Qu Y., Dai B. (2024). Experimental study on flexural performance of ultra-high-performance concrete with recycled aggregate co-modified by nano-silica and steel fiber. Constr. Build. Mater..

[8] Luo L., Jia M., Cheng X. (2024). Experimental study and analytical modeling of tensile performance of ultra-high-performance concrete incorporating modified recycled aggregates. J. Clean. Prod..

[9] Luo L., Sun H., Jia M., Peng B., Li X., Yuan H., Liu G. (2024). Experimental study and theoretical prediction of axial compression behavior in PMC-reinforced CFST columns with void defects. Eng. Struct..

[10] Dong E., Fu S., Wu C., Lv W., Zhang L., Feng Y., Shui Z., Yu R. (2023). Value-added utilization of phosphogypsum industrial by-products in producing green Ultra-High performance Concrete: Detailed reaction kinetics and microstructure evolution mechanism. Constr. Build. Mater..

[11] Tang Y., Feng W., Feng W., Chen J., Bao D., Li L. (2021). Compressive properties of rubber-modified recycled aggregate concrete subjected to elevated temperatures. Constr. Build. Mater..

[12] He Z.-H., Shen M.-L., Shi J.-Y., Yalçınkaya Ç., Du S.-G., Yuan Q. (2022). Recycling coral waste into eco-friendly UHPC: Mechanical strength, microstructure, and environmental benefits. Sci. Total Environ..

[13] Zaid O., Althoey F., Abuhussain M.A., Alashker Y. (2024). Spalling behavior and performance of ultra-high-performance concrete subjected to elevated temperature: A review. Constr. Build. Mater..

[14] Yuan J., Luo L., Zheng Y., Yu S., Shi J., Wang J., Shen J. (2022). Analysis of the working performance of large curvature prestressed concrete box girder bridges. Materials.

[15] Xargay H., Folino P., Nuñez N., Gómez M., Caggiano A., Martinelli E. (2018). Acoustic emission behavior of thermally damaged self-compacting high strength fiber reinforced concrete. Constr. Build. Mater..

[16] Mpalaskas A.C., Kytinou V.K., Zapris A.G., Matikas T.E. (2024). Optimizing building rehabilitation through nondestructive evaluation of fire-damaged steel-fiber-reinforced concrete. Sensors.

[17] Huang K., Xie J., Feng Y., Wang R., Ji J. (2023). Axial impact behaviors of UHPC: The roles of nanomaterials and steel fibres. Constr. Build. Mater..

[18] Mostafa S.A., Faried A.S., Farghali A.A., El-Deeb M.M., Tawfik T.A., Majer S., Abd Elrahman M. (2020). Influence of nanoparticles from waste materials on mechanical properties, durability and microstructure of UHPC. Materials.

[19] Alvi I.H., Li Q., Hou Y., Onyekwena C.C., Zhang M., Ghaffar A. (2023). A critical review of cement composites containing recycled aggregates with graphene oxide nanomaterials. J. Build. Eng..

[20] Zheng Y., Zhang Y., Zhuo J., Zhang Y., Wan C. (2022). A review of the mechanical properties and durability of basalt fiber-reinforced concrete. Constr. Build. Mater..

[21] Iskra-Kozak W., Konkol J. (2021). The impact of nano-Al_2_O_3_ on the physical and strength properties as well as on the morphology of cement composite crack surfaces in the early and later maturation age. Materials.

[22] Luo L., Jia M., Wang H., Cheng X. (2025). Experimental evaluation and microscopic analysis of the sustainable ultra-high-performance concrete after exposure to high temperatures. Struct. Concr..

[23] Liu H., Yang J., Wang X. (2017). Bond behavior between BFRP bar and recycled aggregate concrete reinforced with basalt fiber. Constr. Build. Mater..

[24] Bastami M., Baghbadrani M., Aslani F. (2014). Performance of nano-Silica modified high strength concrete at elevated temperatures. Constr. Build. Mater..

[25] Muzenski S., Flores-Vivian I., Sobolev K. (2019). Ultra-high strength cement-based composites designed with aluminum oxide nano-fibers. Constr. Build. Mater..

[26] Xuan D., Zhan B., Poon C.S. (2018). Thermal and residual mechanical profile of recycled aggregate concrete prepared with carbonated concrete aggregates after exposure to elevated temperatures. Fire Mater..

[27] Wu B., Yu Y., Zhao X.-Y. (2019). Residual mechanical properties of compound concrete containing demolished concrete lumps after exposure to high temperatures. Fire Saf. J..

[28] Wu B., Yu Y., Chen Z., Zhao X. (2018). Shape effect on compressive mechanical properties of compound concrete containing demolished concrete lumps. Constr. Build. Mater..

[29] Zhao H., Wang Y., Liu F. (2017). Stress–strain relationship of coarse RCA concrete exposed to elevated temperatures. Mag. Concr. Res..

[30] Priyadharshini P., Ramamurthy K., Robinson R. (2019). Influence of temperature and duration of thermal treatment on properties of excavated soil as fine aggregate in cement mortar. J. Mater. Civ. Eng..

[31] Khan A.-U.-R., Aziz T., Fareed S., Xiao J. (2020). Behaviour and residual strength prediction of recycled aggregates concrete exposed to elevated temperatures. Arab. J. Sci. Eng..

[32] Chen Z., Zhou C., Li Y., Chen J., Wu B. (2017). Study on the mechanical properties of recycled concrete after high temperature. J. Archit. Struct..

[33] Algourdin N., Pliya P., Beaucour A., Noumowé A., Di Coste D. (2022). Effect of fine and coarse recycled aggregates on high-temperature behaviour and residual properties of concrete. Constr. Build. Mater..

[34] Woodward D. (2009). Chapter 24: Aggregate properties and test methods. ICE Manual of Construction Materials: Volume I: Fundamentals and Theory Concrete.

[35] (2010). Standard Test Method for Flat Particles, Elongated Particles, or Flat and Elongated Particles in Coarse Aggregate.

[36] (1990). Testing Aggregates—Part 112, Methods for Determination of Aggregate Impact Value.

[37] American Society for Testing and Materials (2006). Standard Test Method for Resistance to Degradation of Small-Size Coarse Aggregate by Abrasion and Impact in the Los Angeles Machine.

[38] Mohammed A.A., Khodair Z.T., Khadom A.A. (2020). Preparation and investigation of the structural properties of α-Al_2_O_3_ nanoparticles using the sol-gel method. Chem. Data Collect..

[39] Zhu Y., Hussein H., Kumar A., Chen G. (2021). A review: Material and structural properties of UHPC at elevated temperatures or fire conditions. Cem. Concr. Compos..

[40] Suescum-Morales D., Ríos J.D., Martínez-De La Concha A., Cifuentes H., Jiménez J.R., Fernández J.M. (2021). Effect of moderate temperatures on compressive strength of ultra-high-performance concrete: A microstructural analysis. Cem. Concr. Res..

[41] Zheng W., Li H., Wang Y. (2012). Compressive behaviour of hybrid fiber-reinforced reactive powder concrete after high temperature. Mater. Des..

[42] Wang Y., Li S., Hughes P., Fan Y. (2020). Mechanical properties and microstructure of basalt fibre and nano-silica reinforced recycled concrete after exposure to elevated temperatures. Constr. Build. Mater..

[43] (2019). Test Method for Physical and Mechanical Properties of Concrete.

[44] Pliya P., Hajiloo H., Romagnosi S., Cree D., Sarhat S., Green M. (2021). The compressive behaviour of natural and recycled aggregate concrete during and after exposure to elevated temperatures. J. Build. Eng..

[45] Wei S., Yiqiang C., Yunsheng Z., Jones M. (2013). Characterization and simulation of microstructure and thermal properties of foamed concrete. Constr. Build. Mater..

[46] Silva R.V., De Brito J., Dhir R. (2015). Tensile strength behaviour of recycled aggregate concrete. Constr. Build. Mater..

[47] Mefteh H., Kebaïli O., Oucief H., Berredjem L., Arabi N. (2013). Influence of moisture conditioning of recycled aggregates on the properties of fresh and hardened concrete. J. Clean. Prod..

[48] Dilbas H., Şimşek M., Çakır Ö. (2014). An investigation on mechanical and physical properties of recycled aggregate concrete (RAC) with and without silica fume. Constr. Build. Mater..

